# *In vitro* activity of rifampicin, rifapentine and rifabutin in combination with their 25-deacetyl-metabolites against various *Mycobacterium tuberculosis* lineages

**DOI:** 10.1186/s12941-025-00784-w

**Published:** 2025-02-28

**Authors:** Charlotte Genestet, Chloé Bourg, Elisabeth Hodille, Olivier Bahuaud, Florence Ader, Sylvain Goutelle, Oana Dumitrescu, On behalf of the Lyon TB study group

**Affiliations:** 1 CIRI - Centre International de Recherche en Infectiologie, Ecole Normale Supérieure de Lyon, Université Claude Bernard Lyon-1, Inserm U1111, CNRS UMR5308, Lyon, France; 2 Laboratoire de bactériologie, Hospices Civils de Lyon, Institut des Agents Infectieux, Lyon, France; 3 Service des Maladies infectieuses et tropicales, Hospices Civils de Lyon, Lyon, France; 4 Biometrics and Evolutionary Biology Laboratory, CNRS UMR 5558, Université Lyon 1, Villeurbanne, France; 5 Service de Pharmacie, Hospices Civils de Lyon, GH Nord, Lyon, France; 6 Université Lyon 1, Facultés de Médecine et de Pharmacie de Lyon, Lyon, France

**Keywords:** *Mycobacterium tuberculosis*, Rifamycin, Rifampicin, Rifapentine, Rifabutin, 25-deacetyl-metabolite, Checkerboard assay, Fractional inhibitory concentration

## Abstract

**Objectives:**

Rifamycin agents (rifampicin (RIF), rifapentine (RFP), rifabutin (RFB)) are the cornerstone of tuberculosis (TB) therapy. Rifamycins are metabolized into 25-deacetyl-metabolites, which have been described has active and may contribute to *in vivo* drug effect. However, little is known about the combined effect of rifamycins and their metabolites across different *Mycobacterium tuberculosis* complex (MTBC) lineages.

**Methods:**

This study included 14 MTBC strains representing the main lineages. Minimum inhibitory concentrations (MICs) were determined using microdilution assays for the three rifamycins and their metabolites. A checkerboard assay was used to assess drug interactions, with the fractional inhibitory concentration (FIC) index calculated for synergy or antagonism.

**Results:**

MICs varied across rifamycins, RIF and its metabolite showed the highest MICs, followed by RFP and RFB and their respective metabolites. FIC indices for rifamycin-metabolite combinations indicated additive effects (FIC between 0.5 and 1.25), with no antagonism observed, even at clinically relevant metabolite-to-parent drug ratios, and without impact of MTBC lineage.

**Conclusions:**

Rifamycin metabolites exhibit additive effects with parent drugs, potentially enhancing bactericidal activity. This highlights that rifamycin susceptibility testing should account for both parent drugs and their metabolites, as these metabolites also exhibit antimicrobial activity. Additionally, these findings support further pharmacokinetic/pharmacodynamic studies to optimize TB treatment regimens, particularly in relation to metabolite-to-parent drug ratios in patients.

## Introduction

Tuberculosis (TB), caused by *Mycobacterium tuberculosis* complex (MTBC), remains one of the leading cause of mortality from an infectious agent, accounting for 1.3 million deaths and approximately 10 million new cases worldwide in 2022 [1]. The cornerstone of TB treatment relies on rifamycin-class antibiotics, including rifampicin (RIF), rifapentine (RFP), and rifabutin (RFB). Rifamycins are particularly important in the treatment of TB because of their bactericidal activity against both actively replicating and dormant bacilli [2]. While RIF has been the most widely used rifamycin agent for decades, RFP and RFB are relevant alternatives due to specific pharmacokinetic (PK) and pharmacodynamic (PD) properties. RFB is often used in patients co-infected with HIV due to its lower potential for drug-drug interactions [3]. RFP has a longer half-life compared to RIF and was first considered in the design of twice-weekly dosing regimens [4]. More recently, RFP with daily dosing has been considered in new regimens to increase rifamycin exposure. A 4-month daily rifapentine-based regimen containing moxifloxacin was demonstrated noninferior to the standard 6-month regimen [5].

*In vivo*, rifamycins undergo metabolism, the main metabolites being 25-deacetyl-derivatives (25-d rifamycin). Human PK data indicate average 25-d metabolite to parent concentration ratio of 4 to 38% [6], 67 to 85% [7] and 6 to 18% [8, 9], for RIF, RFP and RFB, respectively. Those metabolites are considered as active, but limited information exists. Specifically, only one study has evaluated the activity of 25-deacetyl-rifapentine (25-dRFP) against different MTBC strains [10]. As both rifamycins and 25-d metabolites co-exist *in vivo*, it is desirable to assess their activity in combination. To date, no research has examined the combined effect of rifamycins and their metabolites across a diverse panel of clinical MTBC isolates, leaving a gap in our understanding of their combined effect. Accordingly, this study aimed to evaluate the activity of RIF, RFP, and RFB and their respective 25-d metabolites, alone and in combination.

## Methods

### Reagents

The rifamycins—RIF, RFP, and RFB—were purchased from Sigma-Aldrich (Sigma-Aldrich, Saint-Quentin-Fallavier, France), while their corresponding metabolites—25-dRIF, 25-dRFP, and 25-dRFB—were sourced from Santa Cruz Biotechnology (Santa Cruz Biotechnology, Heidelberg, Germany). Stock solutions were prepared at 1 mg/mL in DMSO and stored at -20 °C.

### Clinical strains

A total of 14 clinical strains from the MTBC were selected from the TB diagnosis laboratory’s biobank of the Lyon University Hospital. These strains represented the genetic diversity of MTBC: 2 strains from lineage 1, 2 from lineage 2, 2 from lineage 3, 6 from lineage 4 (subtypes T, H, S, X, LAM, and Cameroon), 1 *Mycobacterium africanum* strain, and 1 *Mycobacterium bovis* strain. All experiments were performed in duplicate in 7H9 medium + 10% OADC (oleic acid, albumin, dextrose, catalase) (Becton Dickinson, Pont-de-Calix, France), using an inoculum of 2 × 10^5^ CFU/mL.

### Determination of MIC

Minimum inhibitory concentrations (MICs) were determined using the standard microdilution method previously described [11]. Rifamycin compounds and their metabolites were tested individually against all clinical strains to establish reference MIC values, with tested concentration ranging from 0.004 µg/mL to 4 µg/mL.

### Checkerboard assay and FIC index calculation

To assess the combined effect of rifamycins and their metabolites, the checkerboard assay method was applied [12], with concentrations ranging from 1/32 x MIC to 2 x MIC. The fractional inhibitory concentration (FIC) index was used to quantify interactions, calculated as follows:

FIC = (MIC rifamycin in combination / MIC rifamycin alone) + (MIC metabolite in combination / MIC metabolite alone).

Results were interpreted as follows: FIC < 0.5, synergy; 0.5 ≤ FIC < 1, additive effect; 1 ≤ FIC ≤ 4, no interaction; FIC > 4, antagonism.

The minimum FIC index was evaluated, and the FIC indices were also calculated at different ratios of metabolite to rifamycin, based on the typical metabolite-to-parent drug ratios observed in PK/PD studies.

### Statistical analysis

All statistical analyses were performed using GraphPad Prism^®^ for Windows version 5.02 (GraphPad Software, La Jolla, CA, US). Medians with interquartile ranges were calculated, and comparisons were made using the Friedman test to evaluate differences in MICs and FIC indices across various rifamycin and metabolite combinations.

## Results

**Fig. 1:**
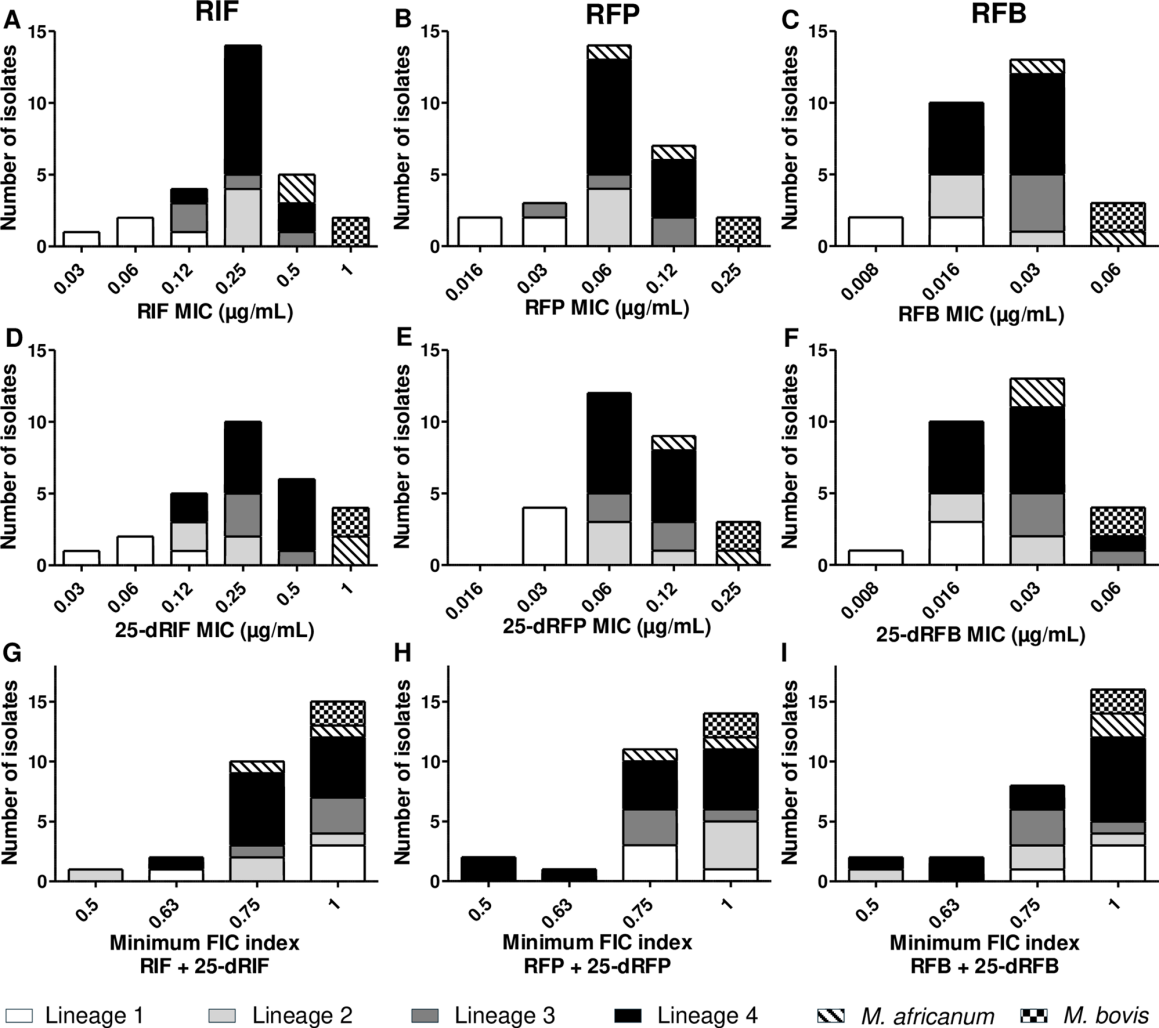
MIC and minimum FIC index of rifamycins and their metabolites

Using standard microdilution method, MICs of rifamycins and their metabolites were evaluated for the 14 MTBC strains selected (Fig. 1). For RIF and 25-dRIF, MICs ranged from 0.03 to 1 µg/mL, with the majority of strains (14/28) displaying values of 0.25 µg/mL. In comparison, MICs for RFP and 25-dRFP were generally lower, ranging from 0.016 to 0.25 µg/mL, with most strains (14/28) showing MIC of 0.06 µg/mL. RFB and 25-dRFB exhibited the lowest MICs, ranging from 0.008 to 0.06 µg/mL, with the majority of values (22/28) at 0.016–0.03 µg/mL. Strains from lineage 1 presented the lowest MICs, while *M. bovis* strain showed the highest values. Notably, there was no significant difference between the MICs of the rifamycins and their corresponding metabolites.

Fourteen clinical MTBC strains were selected to represent the main lineages, lineage 1 (white), lineage 2 (light grey), lineage 3 (dark grey), lineage 4 (black), *M. africanum* (hatched) and *M. bovis* (checkerboard pattern). Minimum inhibitory concentrations (MIC) for rifampicin (RIF, A), rifapentine (RFP, B), rifabutine (RFB, C) and their metabolites 25-deacetyl-rifampicin (25-dRIF, D), 25-deacetyl-rifapentine (25-dRFP, E), 25-deacetyl-rifabutine (25-dRFB, F), were determined by standard microdilution assay. Minimum fractional inhibitory concentration (FIC) index for RIF + 25-dRIF, RFP + 25-dRFP and, RFB + 25-dRFB combinations were determined by checkerboard assay. FIC < 0.5 indicates synergy; FIC between 0.5 and 1 indicates an additive effect; FIC between 1 and 4 indicates no interaction; FIC > 4 indicates antagonism. Experiments were performed in duplicate.

In combination, the minimum FIC indices for RIF + 25-dRIF, RFP + 25-dRFP, and RFB + 25-dRFB were all between 0.5 and 1, indicating additive effects of all combinations (Fig. 1). The results were consistent across different MTBC lineages, with no significant influence of bacterial lineage on the interaction between rifamycins and their metabolites.

**Fig. 2:**
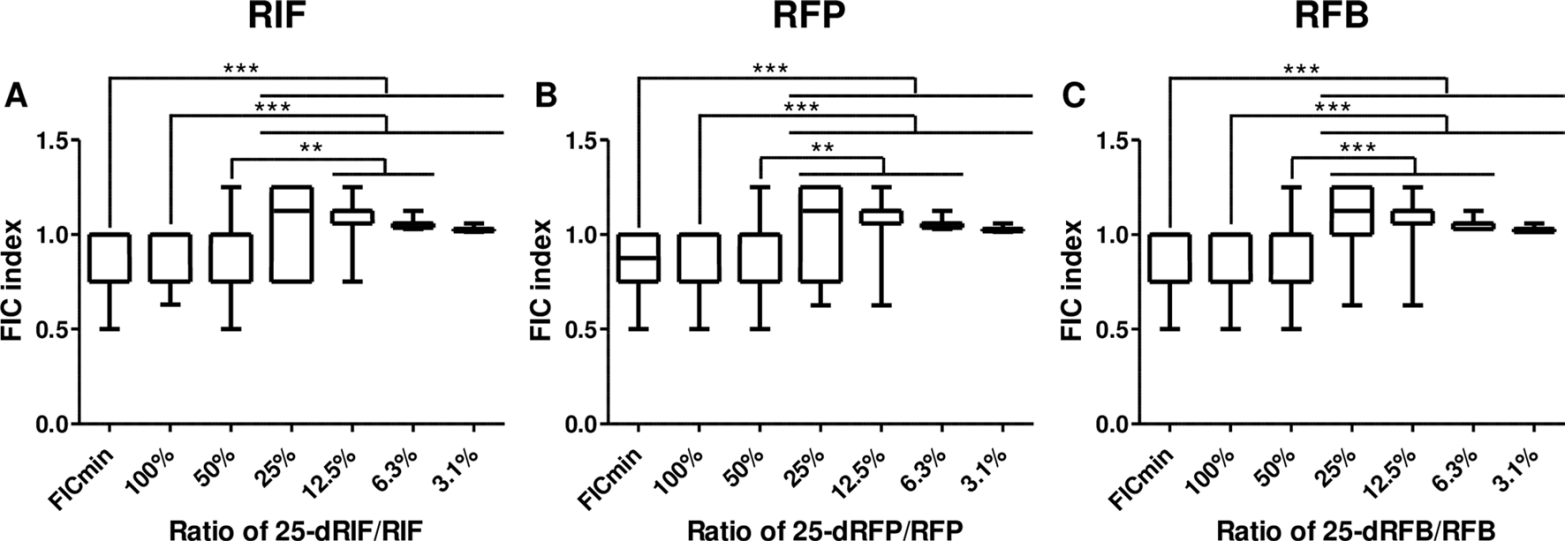
FIC indices at various ratio of metabolite/rifamycin

To further explore the clinical relevance of these interactions, the FIC indices were calculated at different ratios of metabolite to rifamycin (Fig. 2), based on the typical metabolite-to-parent drug ratios observed in PK/PD studies [6–9]. At a ratio of 100% and 50% 25-d-rifamycin/rifamycin, the FIC values were similar to the minimum FIC values, between 0.5 and 1.25. When the ratio of rifamycin to metabolite was reduced to 25% or 12.5%, the FIC values ranged between 0.625 and 1.25, indicating either additive effects or no significant interaction between the parent drug and its metabolite. At the lowest tested ratios of 6.2% or 3.1%, the FIC indices ranged between 1.02 and 1.13, confirming that no antagonism occurs even when the metabolite concentration was minimal. This suggests that the metabolites do not diminish the activity of the parent rifamycins and may still contribute to the overall antibacterial effect.

Fourteen clinical MTBC strains were selected to represent the main lineages (lineage 1, lineage 2, lineage 3, lineage 4, *M. africanum* and *M. bovis*) and rifamycin and metabolite combinations were evaluated using checkerboard assay. Fractional inhibitory concentration (FIC) indices were calculated at different ratios of metabolite to rifamycin, for 25-deacetyl-rifampicin (25-dRIF) + rifampicin (RIF, A), 25-deacetyl-rifapentine (25-dRFP) + rifapentine (RFP, B) and 25-deacetyl-rifabutin (25-dRFB) + rifabutin (RFB, C). These values were compared to the minimum FIC value obtained in each experiment. FIC < 0.5 indicates synergy; FIC between 0.5 and 1 indicates an additive effect; FIC between 1 and 4 indicates no interaction; FIC > 4 indicates antagonism. Experiments were performed in duplicate. Median values were compared using the Friedman test followed by Dunn’s post-hoc test. *p < 0.05, ** *p* < 0.01, *** *p* < 0.001.

## Discussion

The PK and antimicrobial activity of rifamycin metabolites have been overlooked so far. Only a few PK studies reported data on rifamycin metabolites [6–9, 13]. Regarding their activity, *in vitro* data are scarce. This study provides novel insights on the activity of rifamycins (RIF, RFP, RFB) and their metabolites (25-dRIF, 25-dRFP, 25-dRFB) against a panel of clinical strains representing the genetic diversity of the MTBC.

The MIC results confirm the activity of parent drugs, with RFB showing the lowest MIC. The lineage 1 strains were the most susceptible as shown in previous studies, but the precise mechanisms underlying these differences remain to be elucidated [10, 14, 15]. A key finding is that the metabolites display significant activity, with MIC broadly similar to parent rifamycin. The combination of this metabolite with parent rifamycin results in additive effects, with minimum FIC values between 0.5 and 1. Notably, similar FIC values were observed at higher 25-d-rifamycin/rifamycin ratios, reflecting the upper range of metabolite exposure seen in pharmacokinetic studies, suggesting that these additive effects are relevant at clinically observed concentrations. Furthermore, no antagonism was observed, even at low metabolite concentrations, suggesting that these metabolites may contribute to overall treatment efficacy without diminishing the parent drug’s potency.

Our results may have two major implications. First, as both parent and 25-d-metabolites co-exist in patients and even though they had similar antimicrobial activity on the clinical strains tested, it is important to evaluate the activity of both the parent compound and its metabolite to confirm the susceptibility of the tested strains. This approach would allow a more accurate assessment of the antimicrobial activity of rifamycins *in vivo*. Second, PK/PD studies should also investigate more the exposure to both parent and metabolite drugs. PK/PD variability of the metabolites may explain part of the variability that has been attributed to the parent drugs only so far. Notably, the metabolite-to-parent concentration ratio varies significantly, ranging from as low as 4% for some patients treated with RIF to as high as 85% for some patients treated with RFP. These observations highlight the variability between rifamycin agents and between individual patients. Further research is necessary to investigate the correlation between antimicrobial susceptibility testing of rifamycins in combination with their metabolites and clinical activity in patients with TB [6–9, 13].

A limitation of this study is that it did not evaluate potential interactions between rifamycin metabolites and other drugs commonly used in TB treatment regimens. Such interactions could significantly influence the overall therapeutic efficacy and may reveal synergistic or antagonistic effects not captured in this study. Further research is necessary to investigate these combinations and better understand their impact on treatment outcomes.

Overall, this study suggests that these rifamycin metabolites play a meaningful role in the overall antibacterial activity of the rifamycin agents. Future research should further investigate these interactions in clinical settings to optimize therapeutic strategies and improve treatment outcomes.

## Data Availability

Data is provided within the manuscript.

## Abbreviations

25-d-metabolite: 25-deacetyl-rifamycin metabolite
25-dRFB: 25-deacetyl-rifabutin
25-dRFP: 25-deacetyl-rifapentine
25-dRIF: 25-deacetyl-rifampicin
FIC: Fractional Inhibitory Concentration
MIC: Minimum Inhibitory Concentrations
MTBC: *Mycobacterium tuberculosis* complex
PD: Pharmacodynamic
PK: Pharmacokinetic
RFB: Rifabutin
RFP: Rifapentine
RIF: Rifampicin
TB: Tuberculosis
